# Experimental Investigation of Mechanical Properties of Black Shales after CO_2_-Water-Rock Interaction

**DOI:** 10.3390/ma9080663

**Published:** 2016-08-06

**Authors:** Qiao Lyu, Pathegama Gamage Ranjith, Xinping Long, Bin Ji

**Affiliations:** 1 Wuhan University, Wuhan 430072, China; lvqiao@whu.edu.cn (Q.L.); jibin@whu.edu.cn (B.J.); 2 Key Laboratory of Hubei Province for Water Jet Theory & New Technology, Wuhan 430072, China; 3 Deep Earth Energy Laboratory, Department of Civil Engineering, Monash University, Melbourne 3800, Australia; ranjith.pg@monash.edu

**Keywords:** shale, CO_2_-water-rock interaction, mechanical properties, crack propagation, microstructure

## Abstract

The effects of CO_2_-water-rock interactions on the mechanical properties of shale are essential for estimating the possibility of sequestrating CO_2_ in shale reservoirs. In this study, uniaxial compressive strength (UCS) tests together with an acoustic emission (AE) system and SEM and EDS analysis were performed to investigate the mechanical properties and microstructural changes of black shales with different saturation times (10 days, 20 days and 30 days) in water dissoluted with gaseous/super-critical CO_2_. According to the experimental results, the values of UCS, Young’s modulus and brittleness index decrease gradually with increasing saturation time in water with gaseous/super-critical CO_2_. Compared to samples without saturation, 30-day saturation causes reductions of 56.43% in UCS and 54.21% in Young’s modulus for gaseous saturated samples, and 66.05% in UCS and 56.32% in Young’s modulus for super-critical saturated samples, respectively. The brittleness index also decreases drastically from 84.3% for samples without saturation to 50.9% for samples saturated in water with gaseous CO_2_, to 47.9% for samples saturated in water with super-critical carbon dioxide (SC-CO_2_). SC-CO_2_ causes a greater reduction of shale’s mechanical properties. The crack propagation results obtained from the AE system show that longer saturation time produces higher peak cumulative AE energy. SEM images show that many pores occur when shale samples are saturated in water with gaseous/super-critical CO_2_. The EDS results show that CO_2_-water-rock interactions increase the percentages of C and Fe and decrease the percentages of Al and K on the surface of saturated samples when compared to samples without saturation.

## 1. Introduction

Increasing attention has been given to the reduction of the emission of carbon dioxide (CO_2_), which contributes most of the greenhouse effect. Geological storage of CO_2_ is one of the most promising ways to mitigate global warming and climate change [1]. Shale gas reservoirs, which are characterized as having ultra low permeability and high storage potential, are suitable for CO_2_ sequestration [2,3,4].

As CO_2_ is injected into shale reservoirs, it dissolves into waters or brines and changes the acid-base equilibrium which triggers the dissolution and precipitation of minerals [5,6]. Ketzer et al. [7] observed the dissolution of feldspars and calcite cement and the precipitation of dickite, opal and calcite, and reported that the dissolution of Ca and Fe cations limited the precipitation of carbonate. Lu et al. [8] found that concentrations of cations in groundwater presented two trends, and all the concentration variations were dominated by the precipitation of carbonate minerals. Liu et al. [9] experimentally investigated the chemical reactions of shale in CO_2_ and brine saturation. Micro-scale observation revealed the dissolution of carbonates and feldspars, and the precipitation of carbonates and clay minerals. Similar dissolution and precipitation results have been presented by many other scholars [10,11,12,13,14,15].

The mineral dissolution and precipitation also cause changes of mechanical properties (e.g., deformation, strength) and hydrological properties (e.g., permeability and porosity) of rocks, which lead to risks for CO_2_ sequestration [16,17,18,19]. The variation of hydrological properties reflects to the change of microstructures in rocks which, of cause influence the mechanical properties. Bertier et al. [20] found that dissolution of ankerite/dolomite and Al-silicates increased the porosity/permeability of sandstones. Madland et al. [21] studied the mechanical stability of chalk after saturation with CO_2_ gas and carbonate water. The results showed that CO_2_ gas has less effect on chalk’s strength, while carbonate water leads to considerable decrease of strength. Bennion and Bachu [22] investigated the effect of acid gas (CO_2_ and H_2_S) on the relative permeability-displacement character of four kinds of rocks after injection into deep saline aquifers. Bachu and Bennion [23] also found that the capillary pressure, relative permeability and other displacement characteristics for samples in CO_2_-brine systems rely on the in situ conditions of pressure, temperature and water salinity. Farquhar et al. [24] tested the mineral and porosity variations of sandstones and siltstones after reacting in low-salinity water with super-critical carbon dioxide (SC-CO_2_). The results showed that the calcite content decreased from 17 vol % to 15 vol % after reaction, and an increase in porosity of 1.1 vol % CO_2_-water-rock interactions also cause caprock deformation, which results in the change of effective stress and stress-induced permeability [25,26,27,28]. Clearly, the effect of CO_2_-water-rock interactions on the mechanical properties of shale, especially stress behaviors and crack propagations, which lead to CO_2_ leakage in shale reservoirs, is unclear and needs further study.

A variety of shale samples were obtained to react with gaseous/super-critical CO_2_ in the medium of fresh water in several reactors. A group of uniaxial compressive strength (UCS) tests was conducted to obtain the strength variation. The crack propagations and micro-scale characteristics were recorded by acoustic emission (AE) sensors, ARAMIS digital cameras, and SEM, respectively. The purpose of this study is to improve our understanding of gaseous/super-critical CO_2_-water-rock interactions and help us to know more about the risks of CO_2_ sequestration.

## 2. Geological Setting and Geochemical Interactions

### 2.1. Geological Setting

**Figure 1:**
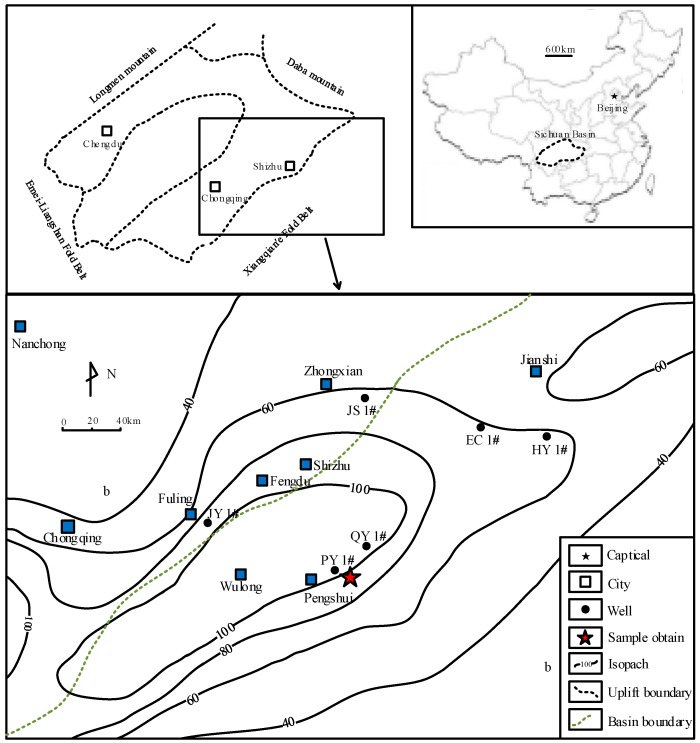
A sketch map of Lower Silurian Longmaxi shale in Southeast Chongqing.

Southeast Chongqing, which is located southeast of the Sichuan Basin, is part of the Upper Yangtze plate. Upper Paleozoic marine black shale (including Cambrian Niutitang Formation and Lower Silurian Longmaxi Formation) in these areas is characterized as having high thickness, suitable burial depth and effective fracture development [29,30]. The thickness of the black shales in south-east Chongqing varies from tens of meters to more than one hundred meters and it is distributed in areas like Fuling, Wulong, Pengshui and Shizhu, as shown in Figure 1. The total organic carbon (TOC) content and equivalent vitrinite reflectance (R_o_) for Longmaxi shale are 2%–4% and 2.5%–3.0%, respectively. The Niutitang shale has a range of TOC content from 3% to 6% and equivalent R_o_ from 3% to 4% [31].

The samples used in the present study were Longmaxi black shales from Shizhu County. Shale outcrops were obtained from an extended shale layer from Pengshui, Chongqing. The mineral compositions obtained using a Bruker AXS D8-Focus X-ray diffractometer (Institute of Rock and Soil Mechanics, Chinese Academy of Sciences, Wuhan, China) are listed in Table 1.

### 2.2. Geochemical Interactions

Due to the dissolution of CO_2_ in water, the weak acid H_2_CO_3_ forms and the pH of the fluid decreases, as described by the following equation:

(1) $${\text{CO}}_{2(aq)}+H_{2}O↔H_{2}{\text{CO}}_{3}↔H^{+}+{\text{HCO}}_{3}^{-}↔2H^{+}+{\text{CO}}_{3}^{2-}$$

When CO_2_ is injected into a shale reservoir, the underground high pressure and temperature cause the pH of the water to decrease dramatically. According to Toews et al. [32], the pH of water in equilibrium with CO_2_ is 2.84 when the temperature and pressure are 40 °C and 7 MPa, respectively. Ultra low pH will result in the dissolution of some minerals, including feldspar, calcite and pyrite [16,24,33].

However, the CO_2_-water-rock reaction is dependent on the environmental conditions [33]. According to Oomole and Osoba [34], higher CO_2_ pressure promotes the dissolution of carbonates, while lower pressure decelerates the dissolution, or accelerates carbonate precipitation. Wigand et al. [35] found that higher pressure and temperature enhance the dissolution of quartz. Iron concentration also influences the reaction [7,36].

Based on the mineral compositions of shale samples (see Table 1), likely reversible reactions are listed in Equations (2)–(5).

(2) $$\begin{array}{l} {\text{KAlSi}}_{3}O_{8}+2H^{+}+H_{2}O↔2K^{+}+{\text{Al}}_{2}{\text{Si}}_{2}O_{5}{\left(\text{OH}\right)}_{4}+4{\text{SiO}}_{2(aq)}\\ \text{K-Feldspar}+2H^{+}+H_{2}O↔2K^{+}+\text{Kaolinite}+4{\text{SiO}}_{2(aq)} \end{array},$$

(3) $$\begin{array}{l} {\text{CaCO}}_{3}+H^{+}↔{\text{Ca}}^{2+}+{\text{HCO}}_{3}^{-}\\ \text{Calcite}+H^{+}↔{\text{Ca}}^{2+}+\text{bicarbonate} \end{array},$$

(4) $$\begin{array}{l} {\text{Fe}}^{2+}+{\text{HCO}}_{3}^{-}↔{\text{FeCO}}_{3}+H^{+}\\ {\text{Fe}}^{2+}+\text{bicarbonate}↔\text{siderite}+H^{+} \end{array},$$

(5) $$\begin{array}{l} {\text{Fe}}^{2+}+{\text{Mg}}^{2+}+{\text{Ca}}^{2+}+3{\text{HCO}}_{3}^{-}↔\text{CaFeMg}{\left({\text{CO}}_{3}\right)}_{3}+3H^{+}\\ {\text{Fe}}^{2+}+{\text{Mg}}^{2+}+{\text{Ca}}^{2+}+\text{bicarbonate}↔\text{mixed}\;\text{carbonate}+3H^{+} \end{array},$$

Equations (2) and (3) present the reversible reactions that influence the dissolution of k-feldspar (Equation (3)) and calcite (Equation (4)). Equations (4) and (5) indicate the likely CO_2_ trapping in mineral through bicarbonate and cations. Kaolinite and pyrite will not dissolve in the CO_2_ equilibrium solution.

## 3. Experimental Methodology

### 3.1. Sample Preparation

Shale blocks were cored parallel to the beddings. The length of each sample was 60 mm, and the diameter was chosen as 30 mm to ensure a height-diameter ratio of 2:1. All the coring and grinding work was finished in the Institute of Rock and Soil Mechanics, Chinese Academy of Science, China, and the experiments were conducted in the Deep Earth Energy Laboratory in the Department of Civil Engineering at Monash University, Melbourne, Australia.

Twelve samples were divided into two groups and saturated in water in equilibrium with CO_2_ at pressures of 7 MPa (gaseous) and 9 MPa (super-critical), respectively. For each group, samples were saturated for different times (10 days, 20 days and 30 days). All the adsorbing conditions had the same temperature of 40 °C. The arrangement of saturation conditions is shown in Table 2. Two samples without saturation were set as the control group. Three slices (with a thickness of 0.4 mm), two of which were saturated together with samples in water with gaseous and super-critical CO_2_ for 30 days, were used for SEM tests.

**Figure 2:**
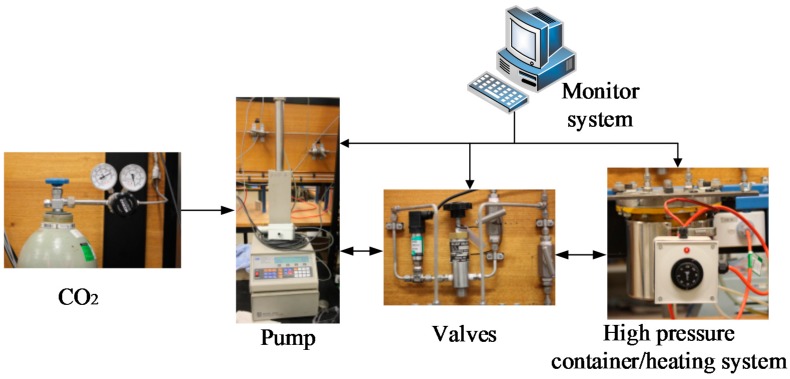
High-pressure adsorbing system.

The saturation system consists of five parts: CO_2_ cylinder, pump, valves, monitor system and container/heating system, as shown in Figure 2. A Model 500D syringe pump (Monash University, Melbourne, Australia) was used to refill CO_2_ into containers. The pressure was controlled by a Teledyne D-series pump controller (Monash University, Melbourne, Australia) with a precision of 1 kPa. The surface of the container was covered by electric resistance wires and the temperature could be adjusted from room temperature to 100 °C.

### 3.2. Testing Arrangement

Fourteen UCS tests were performed on shale samples after CO_2_-water-rock interactions. SHIMADZU AG 9 300 kN compression equipment (Monash University, Melbourne, Australia) was used to conduct the experiments. The loading rate was set at 0.1 mm/min for all the tests. The load and strain were recorded by an advanced data acquisition system. The crack propagations were recorded by acoustic emission (AE) sensors and ARAMIS 3-D technology. The microstructure and X-ray spectra were obtained by a JEOL FE 7001 SEM machine (Monash University, Melbourne, Australia) with a Brucker EDS detector at the Monash MCEM (Monash Centre for Electron Microscopy) center.

## 4. Results and Discussion

### 4.1. Microstructure Alteration after CO_2_-Water-Interaction

**Figure 3:**
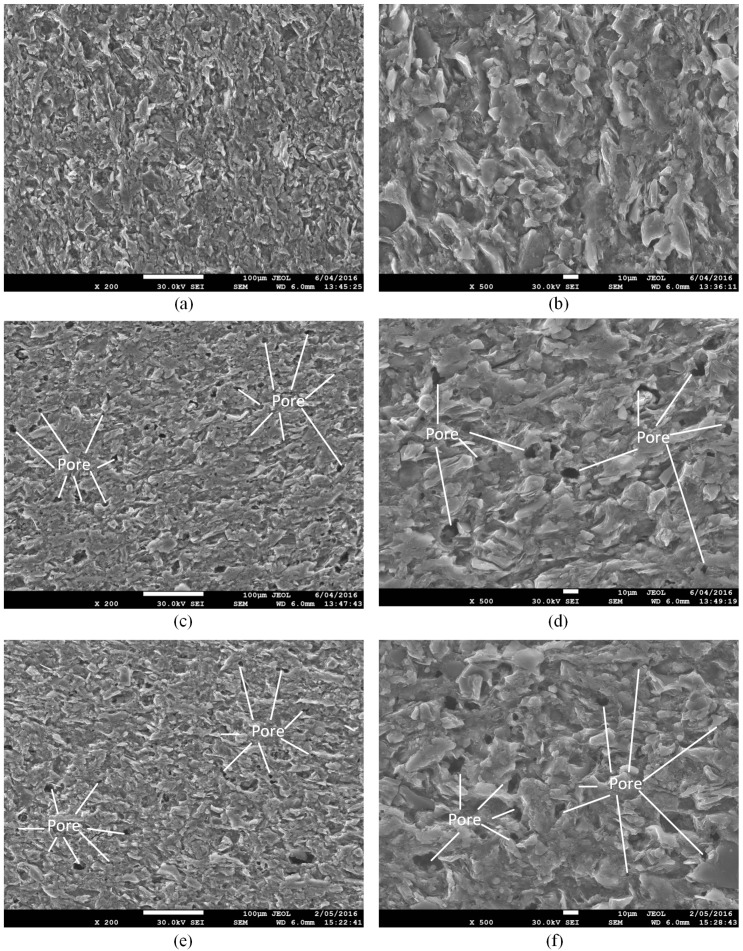
SEM images of of samples without saturation (**a**,**b**); samples with water + gaseous CO_2_ saturation (**c**,**d**) and samples with water + super-critical CO_2_ saturation (**e**,**f**). (**a**,**c**,**e**) have a magnification of 200×; (**b**,**d**,**f**) have a magnification of 500×.

Mineralogical variations associated with microstructure alteration induced by water with gaseous/super-critical CO_2_ was investigated by SEM together with EDS analysis. Figure 3 shows the microstructure of shale samples without any fluid saturation (a,b), samples with water + gaseous CO_2_ (c,d) and water + SC-CO_2_ saturation (e,f). It is clear that the saturation of water with both gaseous and super-critical CO_2_ creates many pores on the surface of the shale slice, possibly because the dissolution of CO_2_ in water decreases the pH of the fluid (Equation (1)), which may accelerate the chemical reactions for mineral dissolution (Equations (2) and (3)) and carbonate precipitation (Equations (4) and (5)). Although SEM can only observe the surface of the sample, we can deduce that, with long-term saturation, water, CO_2_ and ions in the fluids will penetrate into the matrix of the shale and create more pores inside. These pores will create a secondary porosity system, which decreases the strength of the natural pore structure, and the strength of the sample therefore decreases after saturation [37,38]. As SEM analysis can only concentrate on an ultra-small area of the slice’s surface, the difference of the effect of gaseous CO_2_ and SC-CO_2_ on the microstructure of shale samples is minor in the SEM images.

**Figure 4:**
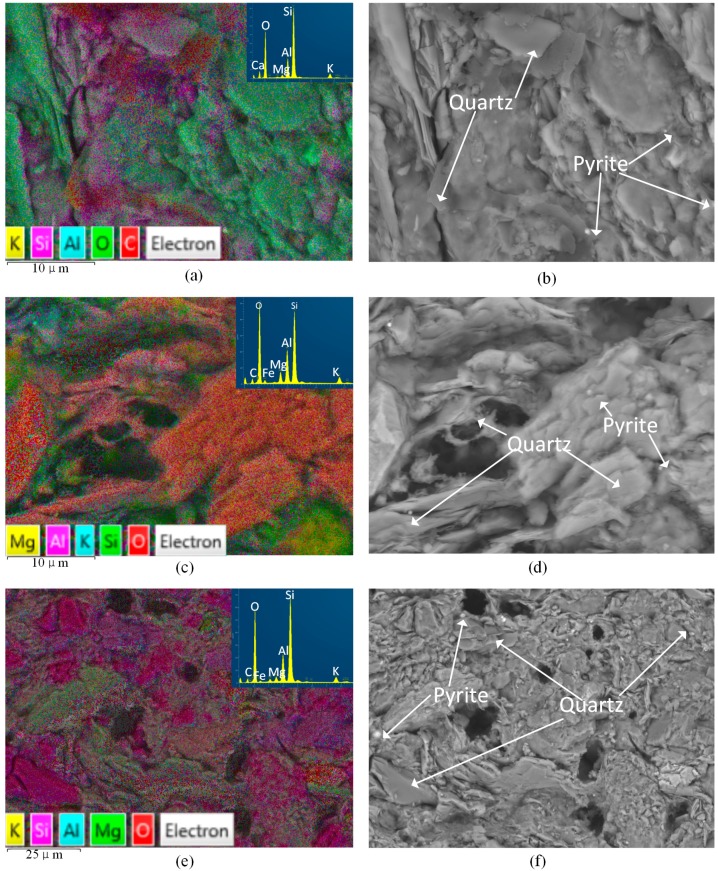
EDS images of samples without saturation and samples with water and gaseous/super-critical CO_2_ saturation. (**a**,**c**,**e**) are chemical mapping and X-ray spectra; (**b**,**d**,**f**) are compacted SEM results.

**Figure 5:**
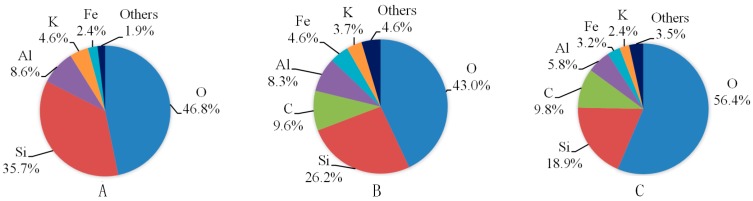
Chemical element composition by EDS analysis. (**A**) is for sample without saturation; (**B**) is for sample with water and gaseous CO_2_ saturation and (**C**) is for sample with water and SC-CO_2_ saturation.

Figure 4 shows the EDS results of shale samples with or without fluid saturation. The X-ray spectra results are shown in Figure 5. For each slice, we observed three different areas and here present the one with a moderate percentage of carbon. From Figure 4 and Figure 5 we can see that oxygen and silica are the first and second highest proportion of all the elements of the three kinds of shale slices. For slices after saturation with water with gaseous CO_2_ and SC-CO_2_, carbon accounts for the third highest percentage of the elements, which are 9.6% and 9.8%, respectively. However, for samples without saturation, the percentage of carbon is negligible. This is mainly because the precipitation of carbonates attaches on the surface of the shale slice. As the gaseous and super-critical CO_2_ applied in the study have similar solubility and pH in water (shown in Table 4), they present similar percentages of carbon. Compared to the spectra of samples without saturation, ions like Al and K decrease and Fe increase in samples with water and CO_2_ saturation. This is caused by the dissolution of minerals, such as K-feldspar.

### 4.2. Effects of CO_2_-Water-Rock Interaction on Mechanical Behaviors

**Figure 6:**
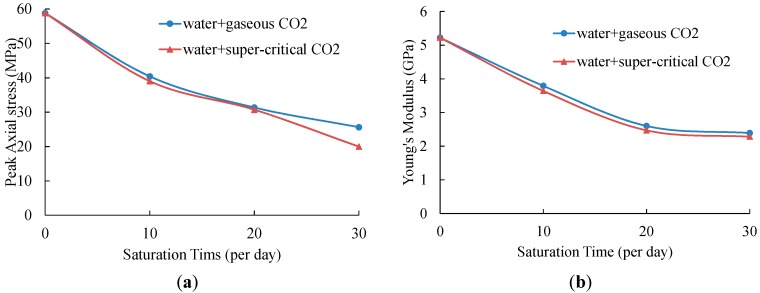
Variation of UCS (**a**) and Young’s modulus (**b**).

The variation of UCS and Young’s modulus of samples without saturation and samples saturated in water absorbed with gaseous and super-critical CO_2_ are shown in Table 3 and Figure 6. The standard deviations of UCS values and Young’s modulus for each saturation condition are minor, except for the two samples saturated in water with SC-CO_2_ for 30 days, which show large UCS variation. Considering the change of strength with saturation time, we chose the higher one for the purposes of discussion. For other groups, the average values are used in all discussions.

According to Table 3 and Figure 6, of all the tested samples, samples without saturation have the highest UCS and Young’s modulus values, of 58.82 MPa and 5.22 GPa, respectively. After 10-day saturation in water with gaseous CO_2_, the UCS value decreases to 40.42 MPa, and the Young’s modulus also shows a reduction of 27.39% to 3.79 GPa. When the saturation time is 20 days, samples show only 31.36 MPa strength and 2.60 GPa Young’s modulus, which is 46.48% and 49.81% respectively lower than samples with no saturation. For 10 more days of saturation, the UCS and Young’s modulus keeps decreasing and the values are smaller than half of that of samples without saturation, at 25.63 MPa and 2.39 GPa, respectively. For shale samples saturated in water with SC-CO_2_, the UCS values show reductions of 33.66%, 47.77% and 66.05% with saturation times of 10 days, 20 days and 30 days, respectively. The Young’s modulus also decreases dramatically from 5.22 GPa to 3.64 GPa (10-day saturation), 2.47 GPa (20-day saturation) and 2.28 GPa (30-day saturation), respectively.

The considerable reductions of strength and Young’s modulus for saturated samples are due to the CO_2_-water-rock interactions coupled with chemical and mechanical effects. When shale samples are saturated in gaseous/super-critical CO_2_ with the medium of fresh water, clays in the rock absorb water resulting in the shale swelling, which causes the decrease of strength and Young’s modulus [39]. According to Heller and Zoback [40] and Luo et al. [41], shale gas, which exists in natural fractures, porous matrices and kerogen, is easier to be replaced by CO_2_ as CO_2_ has better adsorption ability in shale [42]. Therefore, the adsorption of CO_2_ in shale samples will also cause shale swelling and strength decrease [43]. More importantly, the dissolution of CO_2_ in water leads to the chemical reactions for mineral dissolution (Equations (2) and (3)) and carbonate precipitation (Equations (4) and (5)). The dissolution and precipitation process creates pores in the rock, as shown in Figure 3, which changes the microstructure of shale samples. This phenomenon will contribute to the reduction of strength and Young’s modulus. Meanwhile, longer saturation time will cause greater damage on the shale sample, and the strength and Young’s modulus will therefore be lower. This is in accordance with the experimental results.

From Figure 6, we can see that the UCS and Young’s modulus values of samples saturated in water with gaseous and super-critical CO_2_ have the same variation trend with saturation time. However, with the same saturation time, both of the values of samples soaked in water + SC-CO_2_ fluids are smaller than those of samples soaked in water + gaseous CO_2_ fluids. The small discrepancies of strength and Young’s modulus are mainly caused by the difference of properties between gaseous CO_2_ and SC-CO_2_, as shown in Table 4. SC-CO_2_ has higher density, viscosity, thermal conductivity and dissolution ability in water than gaseous CO_2_. The pH of water dissoluted with these two fluids is similar, that of SC-CO_2_ based water being 2.83 and that of gaseous CO_2_ based water being 2.84. These differences overall contribute to the difference in results. Moreover, water, CO_2_ and ions under super-critical saturation conditions will more easily penetrate into shale samples than under gaseous saturation conditions because of the 2 MPa higher confining pressure. 

Another important mechanical characteristic of reservoir rock is the brittleness index (*BI*). The brittleness index can be obtained by many methods, including mechanical analysis, energy analysis and mineral composition analysis [46]. In the present study, mechanical analysis was used for the calculation of the brittleness index (*BI*). It is defined by the following equation [47].

(6) $$BI=\frac{\text{reversible}\;\text{strain}}{\text{total}\;\text{strain}}$$

**Figure 7:**
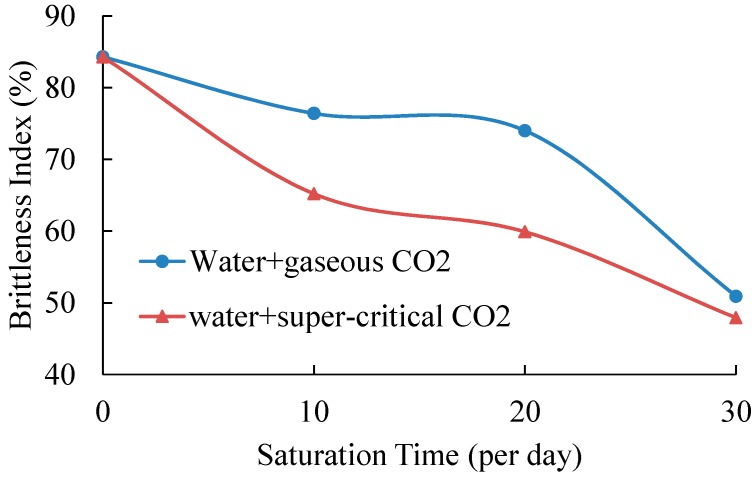
Variations of brittleness index for shale samples.

Table 5 shows the brittleness index of all tested samples and Figure 7 presents the variations of brittleness index with saturation time. The standard deviations of samples without saturation and samples saturated in gaseous/super-critical CO_2_ for 10 and 20 days are minor, varying from 1.2% to 3.8%. However, when the saturation time is 30 days, the samples saturated under gaseous conditions have a high standard deviation of 7.5%, and samples saturated under super-critical conditions have only one value (the other one is excluded because of the ultra-low UCS value). This means that samples with a longer saturation time will have larger variations in the brittleness index, which is caused by chemical-mechanical effects. However, the average value is still more reasonable for analysis.

According to Table 5 and Figure 7, samples without saturation have the highest brittleness index of 84.3%, which is consistent with the mineralogical analysis of samples that contain a high percentage of rigid components. After 10-day saturation, the values of samples in water with gaseous/super-critical CO_2_ decrease to 76.4% and 65.2%, respectively. This is mainly because the adsorption of water and CO_2_ and the chemical reactions of shale and fluids increase the plasticity and toughness. When the saturation time is 20 days, the brittleness index of both the two saturation conditions continues to increase. For samples saturated under gaseous conditions, the brittleness is 74.0%, slightly smaller than that of samples saturated under the same conditions for 10 days. Samples with SC-CO_2_ and water saturation present a much lower brittleness index of 59.9%. Compared to the results of 10-day saturation, 20-day saturation may create more cracks and pores in shale samples, which cause shale samples to have higher plasticity. When the saturation time is extended to 30 days, both kinds of saturated samples present higher axial strains than those with a shorter saturation time before failure, and their brittleness index decreases to 50.9% and 47.9%, respectively. The discrepancy of physical properties (Table 4) between gaseous and super-critical CO_2_ has less effect on shale strength and Young’s modulus. However, their influence on shale’s brittleness index is considerable. Samples with SC-CO_2_ and water saturation present a lower brittleness index than that of samples saturated in gaseous CO_2_ and water fluids for all three saturation times. This means that the beneficial properties of SC-CO_2_ decrease shale’s brittleness and increase its plasticity.

### 4.3. Effects of CO_2_-Water-Rock Interaction on Crack Propagation

**Figure 8:**
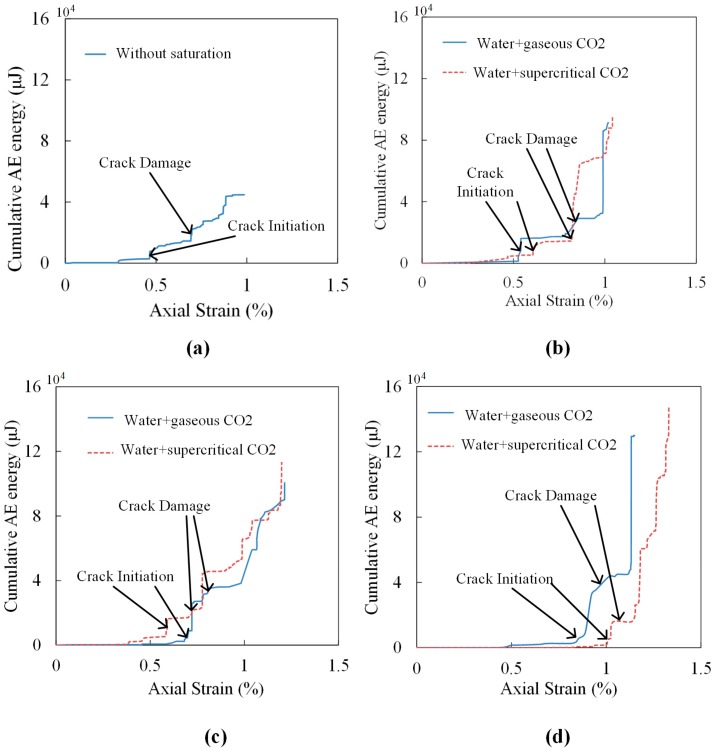
Variation of cumulative AE energy with axial strain for sample without saturation (**a**), samples saturated in gaseous and super-critical CO_2_ for 10 days (**b**); 20 days (**c**) and 30 days (**d**).

With the benefits of high sensitivity and non-destructive monitoring, AE analysis is a widely used method to investigate the stages of crack closure, crack initiation and crack damage in rock mechanics studies and engineering applications [37,48]. The crack closure stage is characterized by very small AE energy, which is released by seating and loading adjustment. With the increase of axial load, stable crack growth or dilation occurs and the AE energy increases gradually, leading to the beginning of crack initiation. When rock samples reach the crack damage point, the AE energy increases drastically and the unstable crack growth creates considerable damage and samples finally fail. Hence, AE analysis provides an additional way to manifest rock mechanics during UCS tests. Figure 8 shows the variation of cumulative AE energy with axial strain for all kinds of saturated samples. As each group has two samples, here we present only one of the AE results for each saturation condition. The cumulative AE energy and axial stress for samples on both crack initiation and crack damage and the peak cumulative AE energy are listed in Table 6.

According to Figure 8 and Table 6, samples with longer saturation time show higher axial strain when failure occurs. This is mainly because of the swelling of samples caused by water and CO_2_ adsorption, and the occurrence of pores and cracks causes samples to have higher strain variations. This also indicates that CO_2_ saturation decreases sample brittleness and increases its plasticity. When samples reach the crack initiation point, their values of cumulative AE energy are low, accounting for a small amount of the peak value ranging from 1.4% to 6.4%. A sample without saturation has the highest axial stress at this point, which is 23.36 MPa. The axial stress for samples saturated in gaseous CO_2_ and water increase with the increase of saturation time, and the corresponding proportion of peak axial stress increases from 31.6% to 77.2%. For samples saturated under super-critical conditions, the axial stress decreases with the extension of saturation time. However, the proportion of axial stress on peak stress increases with increased saturation time, meaning that samples after gaseous/super-critical CO_2_ and water saturation require a higher percentage of peak UCS value to reach the crack initiation when the saturation time is longer. From Figure 8, we can also observe that samples saturated under gaseous conditions reach crack initiation faster than under super-critical conditions when the saturation durations are 10 days and 30 days. In contrast, for 20-day saturation, the trend is reversed. Samples saturated in water with SC-CO_2_ reach the crack damage point earlier than samples saturated in water with gaseous CO_2_ in 20 days. However, when saturation durations are 10 days and 30 days, the crack damage points are almost the same for both kinds of saturated samples. The reason for this difference is mainly because shale samples are anisotropic. When samples reach to the crack damage point, the cumulative AE energy increases to a higher level, ranging from 10.6%–38.2% on peak cumulative AE energy.

According to Figure 8, Table 3 and Table 6, peak cumulative AE energy shows a negative correlation with the UCS values. A sample without saturation has the lowest peak cumulative AE energy of 44,802 μJ. For samples saturated in water with gaseous/super-critical CO_2_, the peak cumulative AE energy increases with increasing saturation time. Specifically, with 10 days’ saturation, the values for samples under gaseous and super-critical conditions doubled and reached 91,227 μJ and 94,840 μJ, respectively. After 20- and 30-day saturation, the values of peak cumulative AE energy were 100,564 μJ and 130,037 μJ for samples under gaseous conditions, and 116,068 μJ and 147,862 μJ for samples under super-critical conditions, respectively. This phenomenon is mainly caused by several reasons. Firstly, the adsorption of water and CO_2_ increases the conductivity of AE emissions. Meanwhile, the swelling caused by water and CO_2_ adsorption creates more artificial fractures. The propagation of these fractures creates more AE energy. In addition, carbonates created by the precipitation process are crushed during the UCS tests and generate acoustic emissions. More importantly, according to the SEM results in Figure 3, the numerous pores produced by chemical reactions decrease the brittleness and increase the plasticity of samples. Therefore, after the peak strength point, samples with CO_2_ saturation can still bear load and create AE energy.

## 5. Conclusions

A series of UCS tests was conducted on samples without saturation, and samples saturated in water with gaseous/super-critical CO_2_ for different periods of time. AE and SEM analyses were performed to evaluate the influence of gaseous/super-critical CO_2_-water-rock interactions and different saturation times on the mechanical properties of Chinese black shale. Several detailed conclusions can be drawn as follows:

Gaseous/super-critical CO_2_-water rock interactions weaken the mechanical properties of shale, and the reductions of UCS, Young’s modulus and brittleness index are closely related to the saturation time. With a saturation time of 10 days, water with gaseous CO_2_ can cause reductions of 31.28% of UCS and 27.39% of Young’s modulus, while water with SC-CO_2_ causes UCS and Young’s modulus decreases of 33.66% and 30.27%, respectively. By extending the saturation time to 30 days, the UCS and Young’s modulus show reductions of 56.43% and 54.21% for water with gaseous CO_2_ saturation, and 66.05% and 56.32% for water with SC-CO_2_ saturation, respectively. The brittleness also decreases with increased saturation time. After 30-day saturation, the value decreases from 84.3% for samples without saturation to 50.9 for samples saturated under gaseous conditions, to 47.9% for samples saturated under super-critical conditions. The decrease of mechanical properties is partially due to the CO_2_-water-rock interactions, which dissolute mineral components and precipitate carbonates. Because of the small gap of physical properties, the effect of the different phase of CO_2_ on shale’s UCS and Young’s modulus is minor.

The saturation of water with gaseous/super-critical CO_2_ increases the total cumulative AE energy of shale samples, which shows a positive correlation with saturation time. Longer saturation time creates higher axial strain when failure occurs. For samples without saturation, the peak cumulative AE energy is 44,802 μJ. After 10 days’ saturation in water with gaseous CO_2_, the value increases to 91,227 μJ, which is smaller than for 30-day saturation. Samples saturated in water with SC-CO_2_ show a similar trend. However, the peak cumulative AE energy for super-critical saturation is slightly higher than that under gaseous saturation when the saturation time is the same.

Based on the SEM results, many pores occur on the surface of shale samples after 30-day saturation in water with gaseous/super-critical CO_2_. EDS analysis shows that CO_2_-water-rock interactions increase the percentages of C and Fe and decrease the percentages of K and Al on the surface of saturated samples. The changes of microstructure and chemical elements indicate the decrease in mechanical properties of saturated samples.

CO_2_ sequestration is a long-term program. However, in this study, we can only propose some short-term rules for CO_2_-water-rock interactions. Therefore, for a better understanding of the effects of CO_2_-water-rock interactions on shale’s mechanical properties, experiments with a much longer saturation time are necessary.

## Figures and Tables

**Table 1:** Mineral composition of shale samples.

Mineralogical Analysis	Value (%, *w*/*w*)	Chemistry
Quartz	58.38	SiO_2_
Potassium Feldspar	14.57	KAlSi_3_O_8_
Muscovite	5.57	KAl_2_(Si_3_AlO_10_)
Calcite	9.54	CaCO₃
Pyrite	4.08	FeS_2_
Smectite	3.43	(Al,Mg)_2_[Si_4_O_10_](OH)_2_·nH_2_O
Illite	1.42	K_1.5_Al_4_(Si_6.5_Al_1.5_O_20_)(OH)_4_
Annite	1.41	--
Kaolinite	1.00	Al_2_Si_2_O_5_(OH)_4_
Braunite	0.37	--

**Table 2:** Saturation arrangements.

Experimental Stage	Stage Duration (Days)	Pressure (MPa)	Temperature (°C)	Number of Samples
Without saturation	--	--	--	2
Gaseous	10	7	40	2
	20	7	40	2
	30	7	40	2
Super-critical	10	9	40	2
	20	9	40	2
	30	9	40	2

**Table 3:** Values of uniaxial compressive strength (UCS) and Young’s modulus (E) for all tested samples.

Specimen		UCS, MPa	Average UCS, MPa (Standard Deviation, MPa)	∆UCS, %	Young’s Modulus, GPa	Average *E*, GPa (Standard Deviation, GPa)	∆*E*, %
Without saturation	NO. 1	56.33	58.82 (2.49)	—	5.41	5.22 (0.20)	—
	NO. 2	61.31			5.02		
Gaseous CO_2_ + water	10 days						
	NO. 1	37.85	40.42 (2.57)	31.28%	3.61	3.79 (0.18)	27.39%
	NO. 2	42.98			3.96		
	20 days						
	NO. 1	29.55	31.36 (1.81)	46.68%	2.44	2.60 (0.16)	49.81%
	NO. 2	33.16			2.76		
	30 days						
	NO. 1	23.49	25.63 (2.14)	56.43%	2.23	2.39 (0.16)	54.21%
	NO. 2	27.76			2.54		
Super-critical CO_2_ + water	10 days						
	NO. 1	37.42	39.02 (1.60)	33.66%	3.45	3.64 (0.19)	30.27%
	NO. 2	40.62			3.83		
	20 days						
	NO. 1	27.55	30.72 (3.17)	47.77%	2.25	2.47 (0.22)	52.68%
	NO. 2	33.89			2.69		
	30 days						
	NO. 1	19.97	19.97	66.05%	2.28	2.28	56.32%
	NO. 2	9.35 (excluded)			--		

**Table 4:** Comparison of physical properties of gaseous CO_2_ (7 MPa, 40 °C) and SC-CO_2_ (9 MPa, 40 °C) and their dissolution abilities in water [32,44,45].

Properties	Phase	Density kg/m^3^	Viscosity μPa·s	Thermal Conductivity W/m·K	Dissolution in Water dm^3^/kg	pH of Water
Gaseous CO_2_	Vapor	198.55	19.288	0.031	26.23	2.84 ± 0.02
SC-CO_2_	Super-critical	492.75	35.360	0.071	27.81	2.83 ± 0.02

**Table 5:** Brittleness index for samples with different saturation conditions.

Saturation Condition	Saturation Time/Days							
	0		10		20		30	
	*BI* %	Average % (Standard Deviation)	*BI* %	Average % (Standard Deviation)	*BI* %	Average % (Standard Deviation)	*BI* %	Average % (Standard Deviation)
Without saturation	83.1	84.3 (1.2)	—	—	—	—	—	—
	85.4		—		—		—	
Gaseous	—	—	79.8	76.4 (3.4)	75.6	74.0 (1.7)	43.4	50.9 (7.5)
	—		73.0		72.3		58.3	
Super-critical	—	—	68.2	65.2 (3.0)	62.5	59.9 (2.6)	47.9	47.9
	—		62.2		57.3		—	

**Table 6:** Crack propagation threshold values of stress and cumulative energy release from AE results.

Saturation Condition		Crack Initiation (% of peak)		Crack Damage (% of peak)		Peak Cumulative AE Energy μJ
		Cumulative AE Energy μJ (% of peak)	Axial Stress MPa (% of peak)	Cumulative AE Energy μJ (% of peak)	Axial Stress MPa (% of peak)	
Without saturation		2864 (6.4)	23.36 (39.7)	14,511 (32.4)	44.15 (75.1)	44,802
Gaseous	10 days	1377 (1.5)	12.77 (31.6)	17,518 (19.2)	26.52 (65.6)	91,227
	20 days	2347 (2.3)	15.76 (50.3)	38,369 (38.2)	21.93 (70.0)	100,564
	30 days	3578 (2.8)	19.79 (77.2)	46,995 (36.1)	22.09 (86.2)	130,037
Super-critical	10 days	5306 (5.6)	18.86 (48.3)	14,526 (15.3)	31.57 (80.9)	94,840
	20 days	5306 (4.6)	16.40 (53.4)	23,395 (20.2)	26.48 (86.2)	116,068
	30 days	2039 (1.4)	14.29 (71.5)	15,745 (10.6)	19.13 (95.8)	147,862
